# Benefits and costs of ecological restoration: Rapid assessment of changing ecosystem service values at a U.K. wetland

**DOI:** 10.1002/ece3.1248

**Published:** 2014-09-23

**Authors:** Kelvin S-H Peh, Andrew Balmford, Rob H Field, Anthony Lamb, Jennifer C Birch, Richard B Bradbury, Claire Brown, Stuart H M Butchart, Martin Lester, Ross Morrison, Isabel Sedgwick, Chris Soans, Alison J Stattersfield, Peter A Stroh, Ruth D Swetnam, David H L Thomas, Matt Walpole, Stuart Warrington, Francine M R Hughes

**Affiliations:** 1 Conservation Science Group, Department of Zoology, University of CambridgeDowning Street, Cambridge, CB2 3EJ, U.K; 2 Institute for Life Sciences, University of SouthamptonUniversity Road, Southampton, SO17 1BJ, U.K; 3 RSPB Centre for Conservation Science, RSPB, The LodgeSandy, Bedfordshire, SG19 2DL, U.K; 4 BirdLife InternationalWellbrook Court, Girton Road, Cambridge, CB3 0NA, U.K; 5 United Nations Environment Programme World Conservation Monitoring CentreHuntingdon Road, Cambridge, CB3 0EL, U.K; 6 National Trust, Wicken Fen National Nature ReserveLode lane, Wicken, Cambridgeshire, CB7 5XP, U.K; 7 Centre for Ecology and HydrologyMaclean Building, Crowmarsh Gifford, Wallingford, OX10 8BB, U.K; 8 Centre for Landscape and Climate Research and Department of Geography, University of LeicesterUniversity Road, Leicester, LE1 7RH, U.K; 9 Botanical Society of Britain and Ireland, Botany Department, The Natural History MuseumCromwell Road, London, SW7 5BD, U.K; 10 Department of Geography, School of Sciences, Staffordshire University, Science CentreLeek Road, Stoke-on-Trent, ST4 2DF, U.K; 11 Animal and Environment Research Group, Department of Life Sciences, Anglia Ruskin UniversityCambridge, CB1 1PT, U.K

**Keywords:** Arable production, biodiversity conservation, ecosystem services, flood protection, global climate change mitigation, nature-based recreation, wetland restoration

## Abstract

Restoration of degraded land is recognized by the international community as an important way of enhancing both biodiversity and ecosystem services, but more information is needed about its costs and benefits. In Cambridgeshire, U.K., a long-term initiative to convert drained, intensively farmed arable land to a wetland habitat mosaic is driven by a desire both to prevent biodiversity loss from the nationally important Wicken Fen National Nature Reserve (Wicken Fen NNR) and to increase the provision of ecosystem services. We evaluated the changes in ecosystem service delivery resulting from this land conversion, using a new Toolkit for Ecosystem Service Site-based Assessment (TESSA) to estimate biophysical and monetary values of ecosystem services provided by the restored wetland mosaic compared with the former arable land. Overall results suggest that restoration is associated with a net gain to society as a whole of $199 ha^−1^y^−1^, for a one-off investment in restoration of $2320 ha^−1^. Restoration has led to an estimated loss of arable production of $2040 ha^−1^y^−1^, but estimated gains of $671 ha^−1^y^−1^ in nature-based recreation, $120 ha^−1^y^−1^ from grazing, $48 ha^−1^y^−1^ from flood protection, and a reduction in greenhouse gas (GHG) emissions worth an estimated $72 ha^−1^y^−1^. Management costs have also declined by an estimated $1325 ha^−1^y^−1^. Despite uncertainties associated with all measured values and the conservative assumptions used, we conclude that there was a substantial gain to society as a whole from this land-use conversion. The beneficiaries also changed from local arable farmers under arable production to graziers, countryside users from towns and villages, and the global community, under restoration. We emphasize that the values reported here are not necessarily transferable to other sites.

## Introduction

Restoration and safeguarding of ecosystems that provide essential ecosystem services (including degraded land) have been recognized by the international community as important means to enhance and maintain biodiversity and ecosystem services, as articulated in Aichi Targets 14 and 15 of the Strategic Plan for Biodiversity 2011–2020 agreed by parties to the Convention on Biological Diversity in October 2010 (CBD 2010). As investments in implementing the Strategic Plan accelerate, governments need information on the relative costs and benefits of particular actions, including ecological restoration, needed to achieve these targets (CBD 2012). In this paper, we assume that for the purposes of valuing ecosystem services, ecosystems can be defined spatially and temporally and use the term ecosystem services to mean the benefits that people receive from ecosystems.

Ecological restoration is usually carried out to benefit biodiversity. There is increasing interest in its effects on ecosystem services, although both may be lower in restoration sites than in the natural habitats that previously existed there (Palmer and Filoso 2009; Rey Benayas et al. 2009). A meta-analysis of 621 restored wetlands shows poor recovery of both biological structures (e.g., plant assemblages) and functioning (e.g., carbon storage), which remain 26% and 23% lower, respectively, than in reference sites (Moreno-Mateos et al. 2012). Irreversible damage to previous ecosystems can explain this discrepancy, although trade-offs between biodiversity and ecosystem services may change through time after restoration starts (Bullock et al. 2011). Where restoration projects emphasize the establishment of ecosystem processes, biodiversity outcomes are less predictable in space and through time (Hughes et al. 2011), but may also more easily achieve ecosystem service gains than projects that are more prescriptive in their spatial planning of habitats and related species targets (Fisher et al. 2011; Perring et al. 2013).

At the Wicken Fen Vision project in Cambridgeshire, U.K., conversion of drained arable land to restored wetland is being carried out by the National Trust, a nongovernmental organization that owns the site (National Trust 2009). Some local councilors and farmers have argued that loss of food production is not in the national or local interest (East Cambridgeshire District Council 2011). In order to elucidate the trade-offs at the center of this debate, we carried out a comparative assessment of the ecosystem services at both the wetland restoration site and on adjacent arable land. To achieve this, we used the Toolkit for Ecosystem Service Site-Scale Assessment (TESSA), a framework for rapid assessment of ecosystem service provision by a site of interest in its current state and in its most likely alternative state (Peh et al. 2013).

## Materials and Methods

### Study area

**Figure 1:**
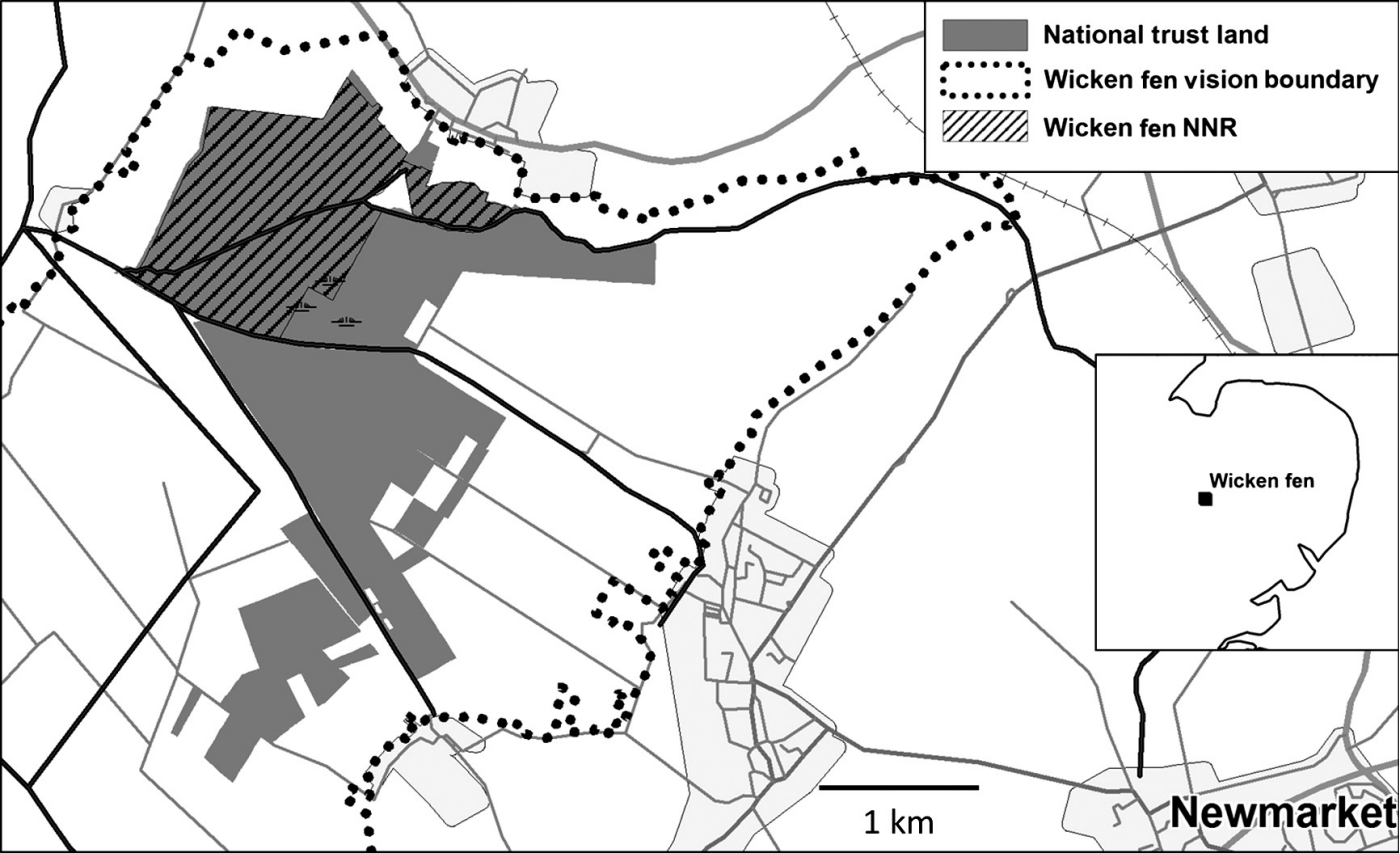
Location of Wicken Fen NNR and the Wicken Fen Vision project land used in this study. Continuous gray area represents restored wetland; adjacent white areas represent arable farmland. (redrawn from Hughes et al. 2011).

The fenland basin of East Anglia in the UK is used for intensive arable agriculture on remnant peat soils of what was once a vast floodplain wetland of about 3850 km^2^ (Moore 1997). Major drainage during the 17th and 19th centuries left only four areas of the original undrained fen wetland, covering just 7.13 km^2^ (0.18%) between them (Rowell, 1997). One of these, Wicken Fen NNR (52°18′24N, 0°16′51E), includes undrained alkaline peats up to four meters in depth and supports seminatural, biodiverse, alkaline fen habitats (Mountford et al. 2005) (Fig.1). Despite its small size (170 ha), it has over 8000 species, many of them rare fen specialist invertebrates. Extinction of some of these rare species is thought to be related to Wicken Fen NNR's small size and inadequate inputs of base-rich groundwater (Colston and Friday 1999). Therefore, some of the adjacent farmland was purchased in 1993 by the National Trust (at market prices) and subsequently converted to a mosaic of wetland and terrestrial habitats.

This initiative has since expanded into a landscape-scale habitat creation project called the Wicken Fen Vision, which is intended to grow over the next 100 years to cover 5300 ha. The project was explicitly intended to increase ecosystem service provision as well as to provide new habitats for wildlife, through increased recreation opportunities and reduced rates of soil organic carbon loss (Colston 2003). The project currently covers 770 ha, including Wicken Fen NNR.

The restored land has structurally damaged peat soils of 20- to 80- cm depth (Stroh et al. 2013). Most of the restored area is partially flooded in winter and is managed year-round with low-density semi-feral grazing animals. In addition, some areas in the first few years of restoration are seasonally grazed by domestic livestock belonging to local farmers. The adjacent land is almost all under intensive arable agriculture, as was the wetland site before restoration, growing various annual crops (Cook 2009). The area is hydrologically complex with canalized rivers elevated c. 3 m above the land level because the drained peats have oxidized and shrunk. The ditch system that drains the farmland is c. 3 m below land level, and its water is lifted into the rivers at a pumping station.

### Assessment of ecosystem services

In this study, we used methods from the TESSA toolkit to compare ecosystem service values of the restored wetland with those of the adjacent arable land. We chose this toolkit because it enables the collection of high resolution, site-scale data, relevant to decisions being made at the Wicken Fen Vision, without the need for specialist technical knowledge of the modeling approaches or GIS software typical of most currently available tools such as INVEST (Tallis et al. 2013) or ARIES (Bagstad et al. 2011). The TESSA toolkit also allowed the ecosystem services assessment to be made rapidly with little field work or substantial investment of staff time. This is in part because the toolkit currently provides valuation approaches for only five ecosystem service areas (Global climate regulation, water-related services, harvested wild goods, cultivated goods, and nature-based recreation) and in part because some forms of economic valuation within the toolkit are simplified versions of more complex and difficult valuation techniques. For example, simple measurements of expenditure on travel to a nature reserve and tourism spend at the nature reserve are used instead of more sophisticated revealed preference methods (Bateman et al. 2011) such as the travel cost method (Bockstael and McConnell 2006; Samos Juarez and Bernabeu Canete 2013), resulting in more conservative valuations.

We first assessed the ecosystem service values of a contiguous block of 479 ha of restored wetland. We then used data from immediately adjacent arable land to estimate what the ecosystem service value of this 479 ha block of land would be if it was still under arable cultivation (Fig.1). We convened a meeting of key stakeholders involved at the wetland restoration site including staff from the National Trust, representatives of the U.K. Environment Agency and Natural England who have oversight on flooding, water abstraction, and biodiversity, respectively, university researchers and local volunteers working at the site. This consultation identified the main, readily measured ecosystem services provided by the restored wetland as (1) global climate change mitigation, (2) nature-based recreation, (3) flood protection, and (4) the provision of grazing. Arable production (5) was identified as the key ecosystem service of the arable land, but local stakeholders (residents who use the area recreationally or are local parish councilors or landowners) identified recreational services as important on arable land as well as on the restored wetland. Non-use values such as existence and bequest value were also identified as important but are far less amenable to quantification and so were not assessed here. As data collection was carried out in 2011, all values were estimated in British pounds and converted to US dollars using a yearly average exchange rate for 2011 of 1 GBP = 1.541 USD.

#### Global climate change mitigation

We assessed fluxes of greenhouse gases (CO_2_, CH_4,_ and N_2_O) for the site under the current and alternative land uses, based on appropriate, published, peer-reviewed values and including emissions from soil, plant, and animal sources (Table1). We converted net flux of each gas (in tonnes ha^−1^y^−1^) into tonnes CO_2_ equivalents (CO_2eq_) ha^−1^y^−1^ and summed these to give a net global warming potential (over 100 years – GWP_100_) ha^−1^y^−1^ under each land use (Forster et al. 2007). These values are also expressed as a total value of tonnes CO_2eq_ y^−^^1^ for the whole site. We used the standard convention of positive values indicating net atmospheric warming. Ranges for all values were calculated using the published uncertainties for each flux additively. We estimated a monetary value of overall greenhouse gas fluxes using six estimates of the price of carbon (Table4).

**Table 1:** Emissions factors used in calculations of greenhouse gas fluxes and global warming potential over 100 years, using the following conversion factors (after Forster et al. 2007): CO_2_ = 1, CH_4_ = 25, N_2_O = 298CO_2eq_

State	Flux	Emission Factor (in original units)	GWP_100_ (tCO_2eq_ ha^−1^ or head^−1^ y^−1^)	Source	Notes
Restored wetland	Soil CO_2_	−169 gC m^−2^y^−1^	Min. -6.20	Lloyd (2006)	We used emission factors for dry or periodically wet grassland on peat, obtained at Wicken Fen and on the Somerset Levels (UK) because the restored land at Wicken Fen is surrounded by heavily drained land still in production and high water levels cannot be maintained year round. This differs from the seminatural wet grassland with a consistently high water table described in Couwenberg et al. (2008), and therefore, methane emissions are likely to be low.
		59 gC m^−2^y^−1^	Max. 2.16	Morrison et al. (2012)	
			Min. 0.49		
			Max. 1.49		
	Soil CH_4_	−0.4 nmolCH_4_ m^−2^sec^−1^	Min. −0.05	Levy et al. (2012)	
			Max. 0		
	Animal CH_4_	57kgCH_4_ head^−1^y^−1^± 50%	Cattle 1.54	IPCC (2006)	
		18kgCH_4_ head^−1^y^−1^± 50%	Horse 0.49		
	Animal N_2_O	*1.6kgN_2_O head^−1^y^−1^± 50%	Cattle 0.47 ± 50%	IPCC (2006)	
		0.4kgN_2_O head^−1^y^−1^ ± 50%	Horse 0.11 ± 50%		
Arable land	Soil CO_2_	227.1 ± 46.5 gCO_2_-C m^−2^	Min. 4.17	Bradley (1997) cited in Natural England (2010)	As above, we have used emission factors associated with thin, wasted peat and have separated emissions from oxidation of soil carbon and those due to N_2_O from fertilizer use. The minimum soil CO_2_ figure is derived from Bradley's (1997) global warming potential values for cultivated thin peat, subtracting the N_2_O value from IPCC to allow the site specific rotation values for fertilizer use to be used.
			Max. 11.62	Morrison et al. (2013)	
	Fertilizer N_2_O	**2.1kgN_2_O ha^−1^y^−1^ (range 0.6–10.0)	0.63 (0.18–2.97)	IPCC (2006)	

* Calculated per head N_2_O emissions from manure deposited on pasture using IPCC Tier 1 default emissions factors and equations given in Chapter 10, section 5 of Volume 4 “Agriculture, Forestry and Other Land Use”, 2006 IPCC Guidelines for National Greenhouse Gas Inventories (IPCC 2006).

** Calculated per hectare direct and indirect emissions from mineral fertilizer used on arable crops (combined across all crops) IPCC Tier 1 default emissions factors and equations given in Chapter 11, section 2 of Volume 4 “N_2_O Emissions From Managed Soils, and CO_2_ Emissions From Lime And Urea Application”, 2006 IPCC Guidelines for National Greenhouse Gas Inventories (IPCC 2006).

For arable farmland, we used regionally typical cropping of winter wheat, oil seed rape, and potatoes in a wheat-rape-wheat-potatoes rotation. We calculated annual emissions by subdividing the area using the ratio 2.45 (wheat):0.5 (rape):0.5 (potato) (after Cook 2009) (see Appendix S1). (This is the same as the ratio of 71% cereal cropping and 29% general cropping (by area) used to calculate the value of arable production services). Under arable cultivation, CH_4_ emissions are likely to be negligible (or even to involve a slight uptake, Rydin and Jeglum 2006; Anderson-Teixeira and DeLucia 2010) due to the aerobic nature of the soil environment, so, we considered only CO_2_ emissions from oxidation of soil organic matter, and N_2_O emissions from mineral nitrogen fertilizer addition (see Appendix S1).

#### Nature-based recreation

Economists working on tourism distinguish two main, non-overlapping components of value (reviewed in Wells 1997): direct expenditure by visitors (an element of economic impact, calculated from spending on fees, travel, food, and accommodation) (e.g., Walpole and Goodwin 2000); and consumer surplus (a measure of economic value, estimated as the difference between what visitors would be prepared to pay for a visit and what they actually spend, calculated through a revealed preference technique such as the travel cost method). Most studies assess just one. Given the rapid nature of our assessment, we focused on the more tractable elements of the first type of measure – direct expenditure – and specifically visitor spend at the site itself and in traveling to get there. The amount spent on travel reflects the minimum value a visitor places on a site for recreation, that is, the cost of getting there, and therefore tends to be a conservative value of nature-based recreation (Farber et al. 2002). The amount spent by tourists on, for example, food and accommodation, are also important aspects of their total spend on their recreational experience because they are monetary transactions related to tourism. By adding them to the amount spent on travel, the total measured recreational value becomes less conservative but it remains an incomplete analysis of the recreational value of the site because non-market components have not been included (Wells 1997).

We estimated the value of nature-based recreation from the direct expenditure by visitors to the site including local tourists (“day-trippers”), national, and international tourists. We estimated the annual number of person-visits to the restored wetland from gate entry data combined with a field survey carried out at the two main access points to the study area on 7 days in late summer (August, September, and October), 2011. These 7 days were chosen to represent the different types of “visitor-days” as identified and classified by the National Trust (see Appendix S2). We used a questionnaire survey to obtain information on distance travelled, mode of transport, places visited, expenditure in the shop and café, and likelihood of visiting restored wetland and arable farmland (see Appendix S4).

#### Grazing

Grazing is carried out on some of the most recently acquired restoration wetland through commercially priced agreements with local farmers. No inputs of fertilizer, pesticides, or irrigation are allowed. A total of 316 ha of the 479 ha is managed in this way. We estimated its value as the rental income paid.

#### Flood protection benefit

The low-lying landscape of the Wicken Fen region is at risk of serious floods if river embankments or the pump drainage system fail during periods of high rainfall (Friday and Rowell 1997). Neither the arable farmland nor Wicken NNR have flood storage capacity, but part of the restored wetland at Wicken has been configured to act as a flood storage area for a 1-in-20-year flood event (Convine and Starling 1988). We estimated the total benefit of this as the value of the avoided damage to crops and property (as calculated by Convine and Starling (1988), updated with current information on the value of crops and property) (See Appendix S3).

#### Arable production

We estimated the value of arable production from published economic data on farming in the surrounding region. Crop selection was established based on a land-use survey of the surrounding regions in 2008 (Cook 2009). The mean per hectare output and costs of farming in the region were obtained from summaries of standard farm accounts reported in the annual Farm Business Survey for 2010–2011 (Lang 2011) and were adjusted to exclude items of income and expenditure not directly related to arable production (Table2). In particular, we excluded agricultural subsidies received by farmers (and by the National Trust for its restored land) under the European Union Common Agricultural Policy, as these do not represent a net benefit to society but rather an internal transfer of value from one part of society to another (Bateman et al. 2011). We also excluded miscellaneous farm activities unrelated to the production of crops, and we excluded interest and rental costs relating to the farmland itself (to be consistent with the analysis of the restored wetland; see below). Finally, we included a value for unpaid manual labor (predominantly that of the farmer and spouse) – this is generally omitted from reported costs, but represents a real cost to the production of cultivated goods.

**Table 2:** Calculation of the output and costs attributable to arable production based on financial data presented in Lang (2011) for cereal farms (growing mainly wheat, barley, and oats) in The Fens (the region in which the Wicken Fen Vision land is located) and for general cropping farms (growing mainly onion, oilseed rape, and root crops) in Cambridgeshire. Values for the arable land were derived by weighting the values for cereals and general cropping by their percentage cover (Cook 2009)

Revenue and cost items (2010-11 $ ha^−1^ y^−1^ unless stated)	Cereals	General cropping	Arable land
% cover (weighting factor)	71%	29%	
Total agricultural output^1^	1872	2971	2191
Less: income from miscellaneous activities^2^	(168)	(120)	(154)
Output attributable to arable production	1704	2851	2037
Total management costs	1368	2270	1630
Plus: unpaid labor^3^	133	116	128
Less: net interest and rent^4^	(114)	(227)	(147)
Less: costs of miscellaneous activities^2^	(105)	(88)	(100)
Costs attributable to arable production	1282	2071	1511

1 Excludes subsidies received under the European Union Common Agricultural Policy.

2 Unrelated to arable production.

3 Generally excluded from reported costs but represents a real cost to arable production.

4 Excluded as interest and rental costs of land are also excluded from the analysis of the restored wetland.

#### Restoration and management costs

We obtained information on the one-off capital costs and subsequent annual management costs of the wetland restoration from National Trust staff at Wicken Fen. The one-off costs included land purchase, fencing, and some re-engineering of ditches. The annual management costs included salaries, equipment, veterinary fees, and fence maintenance. Because the land was purchased outright (incurring a one-off, upfront cost), there are no on-going rental or interest costs associated with the land in the management costs of the restored wetland. To ensure a consistent treatment, interest and rental costs were also excluded from the management costs of the farmland, as outlined above.

## Results

### Global climate change mitigation

**Figure 2:**
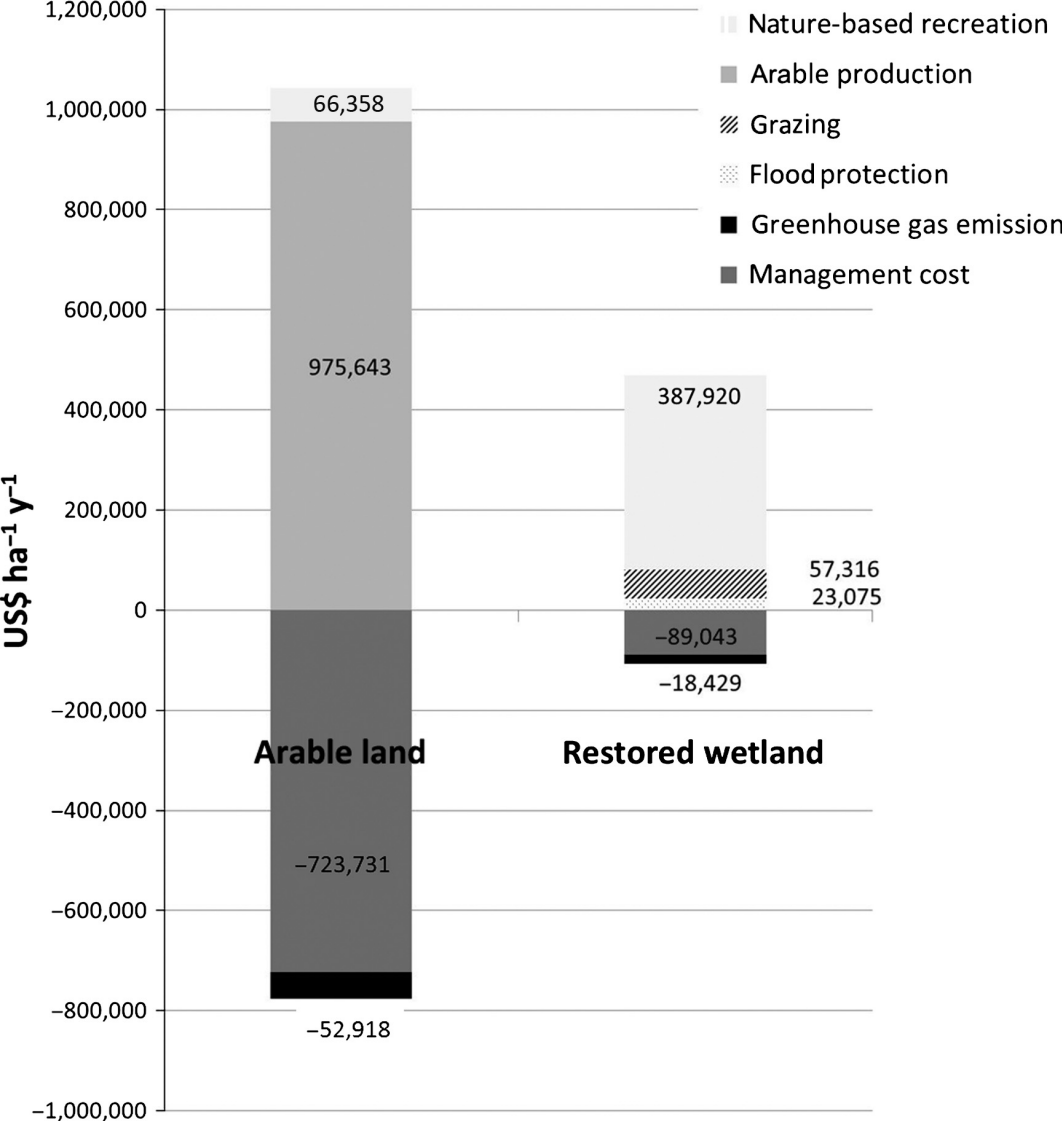
A comparison of the ecosystem service values and management costs in 2011 (in US$for 479ha y−1) of restored wetland and of the same land if returned to arable agriculture.

The total annual global warming potential of the 479 ha of restored wetland in 2011 was estimated at 809 (from −2743 to 1632) tCO_2eq_ y^−1^. (The given range is the minimum and maximum likely emissions value based on the range of emissions factors used in the literature and their published uncertainties and using the highest and lowest reported emissions factors (and associated errors) for each GHG). The majority of this value derives from emissions from the soil and vegetation (either soil carbon oxidation or CH_4_ production, depending on water table) with only a small amount (approximately 255 tCO_2eq_ y^−1^, of CH_4_ and N_2_O) deriving from grazing animals. In contrast, using the same approach, we estimate the arable land emits 2323 (2083–6982) tCO_2eq_ y^−1^. The bulk of this net flux is due to soil carbon oxidation (between 1997 and 5566 tCO_2eq_ y^−1^). A range of economic values for the cost of GHG emissions is presented using six different carbon prices in Table4. We chose the relatively conservative US Government price of $22.78 tonne ^1^CO_2_ (Greenspan Bell and Callan 2011) to give a total value for the cost of GHG emissions of $18,429 ($38 ha^−1^y^−1^) for the restored wetland compared with $52,918 ($110 ha^−1^y^−1^) for the arable land (Table3, Fig.2).

**Table 3:** Net value of all services resulting from the restoration of wetland from arable farmland. *The cost of greenhouse gas emission was based on the US Government CO_2_ value of $22.78 t^−1^ CO_2_, adjusted to 2011

	Restored wetland ($) (479 ha)	Arable land ($) (479 ha)	Difference ($) (479 ha)	Difference ($ha^−1^ y^−1^)
Service flow ($ yr^−1^)				
Flood protection	23,075	0	23,075	48
Grazing	57,316	0	57,316	120
Arable production	0	975,643	975,643	2037
Nature-based recreation	387,920	66,358	321,562	671
Disservice flow ($ yr^−1^)				
Greenhouse gas emission*	18,429	52,918	34,489	72
Management cost ($ yr^−1^)	89,043	723,731	634,688	1325
Net annual benefit ($ yr^−1^)	360,839	265,352	95,487	199
Net annual benefit ($ yr^−1^ ha^−1^)	753	554	199	
Initial Restoration cost ($)	1,110,907	0	1,110,907	2319

**Table 4:** Sensitivity analysis of the costs of greenhouse gas emissions

Sensitivity analysis of the costs of greenhouse gas emission	Restored wetland ($) (479 ha)	Arable land ($) (479 ha)	Difference ($) (479 ha)	Difference ($ha^−1^ y^−1^)
*2011 Carbon dioxide price*				
EU's Emission Trading Scheme (Point Carbon 2012) – $15.31 tonne^−1^ CO_2_	12,386	35,565	23,179	48
US Government (Greenspan Bell and Callan 2011) – $22.78 tonne^−1^ CO_2_	**18,429**	**52,918**	**34,489**	**72**
UK Government (Greenspan Bell and Callan 2011) – $87.01 tonne^−1^ CO_2_	70,391	202,124	131,733	275
Tol (2010) – $32.18 tonne^−1^ CO_2_	26,033	74,754	48,721	102
Stern Review (Stern et al. 2006) – $94.86 tonne^−1^ CO_2_	76,742	220,360	143,618	300
Verified Emission Reductions (Peters-Stanley et al. 2011) – $6.20 tonne^−1^ CO_2_	5016	14,403	9387	20

Sensitivity analysis of the net annual benefits for all services measured				
*2011 Carbon dioxide price*				
Net annual benefits ($ yr^−1^) using EU CO_2_ price	366,882	282,705	84,177	176
Net annual benefits ($ yr^−1^) using US government CO_2_ price	**360,839**	**265,352**	**95,487**	**199**
Net annual benefits ($ yr^−1^) using UK government CO_2_ price	308,887	116,146	192,741	402
Net annual benefits ($ yr^−1^) using Tol (2005) CO_2_ price	353,235	243,516	109,719	229
Net annual benefits ($ yr^−1^) using Stern review CO_2_ price	302,526	97,910	204,616	427
Net annual benefits ($ yr^−1^) using VER CO_2_ price	374,252	303,867	70,385	147

Figures in bold denote those chosen for the overall ecosystem service analysis in Table3.

### Nature-based recreation

We interviewed 892 individuals and counted a total of 2309 visitors (adults and children) of which 28% visited the NNR only, 42% visited the restored wetland only, and 30% visited both areas. Most visitors (93%) were day-trippers from within the region. Domestic and international tourists represented 6% and 1% of the total visitors, respectively. Based on National Trust data, 44,813 people visited the NNR in 2010. Hence, based on the proportions above (collected in 2011), we estimated that in 2010, a total of 32,451 people visited the restored wetland only, of which 30,283 were day-trippers (24,977 adult day-trippers, 5306 children).

From the total reported expenditure of our respondents on travel and in the gift shop and cafe, we estimated the total annual recreational revenue from the people who visited only the restored wetland to be $387,920 ($810 ha^−1^y^−1^; day-trippers contributed $286,666, national tourists $90,107, and international tourists $11,148) (Table3; Fig.2). Hence, the majority of the annual revenue was from the day-trippers. Because some surveys of direct spend on recreation do not include expenditure on food and drink, we have also recalculated the total annual recreational revenue minus the 16% of the total value that was spent in the café to be $324,227 ($677 ha^−1^y^−1^).

Among the day-trippers, 46% of the respondents indicated that they would visit the area if it was arable land. The majority of these were local residents who walk their dogs everyday on local footpaths and who would still use the local footpaths if they were on arable land. No international or national tourists indicated that they would visit the arable land. Based on the expenditure of the day-trippers who would visit the arable land, we estimated a total annual expenditure value of $66,358 ($139 ha^−1^y^−1^) (Table3; Fig.2) for the arable farmland (see Supplementary Information).

### Grazing

Based on the rental agreements between commercial graziers and the National Trust, we estimated the annual net benefit of grazing on the restored wetland as $57,316 ($120 ha^−1^y^−1^) (Table3; Fig.2). There was no grazing associated with the arable land.

### Flood protection benefit

The flood storage capacity of the restored wetland has the potential to protect 2000 ha of farmland in the area (Convine and Starling 1988). Of this, 50 ha would be flooded during a 1-in-20-year flood event and would probably reduce in value for grazing, while the remaining 1950 ha would have a high water table that would only allow cereal crops rather than higher value root crops to be grown (Graves and Morris 2013). In addition, 10 houses would be directly affected by flood damage (Convine and Starling 1988). The total flood cost was estimated at $461,505, comprised of $245,264 due to crop loss or land-use change and $216,241 of damage to homes (see Appendix S3). As the embankment failure is expected to be a 1-in-20-year event, this overall avoided damage cost was then adjusted by a factor of 0.05 to $23,075 per year ($48 ha^−1^y^−1^) (Table3; Fig.2). No flood protection service was provided by the arable land.

### Arable production

Based on Cook (2009), we estimated that crop selection on the arable land would comprise 71% cereal cropping and 29% general cropping (by area). The value of ecosystem services that would be derived from arable production on the 479 ha was estimated to be $975,643 y^−1^ ($2037 ha^−1^y^−1^), offset by management costs (including production costs, labor, machinery and maintenance costs, professional fees, utilities, and property depreciation) of $723,731 y^−1^ ($1511 ha^−1^y^−1^) (Table2). There is no arable production derived from the current restored wetland.

### Restoration and management costs

The on-going management cost of the wetland was estimated to be a total of $89,043 y^−1^ ($186 ha^−1^y^−1^) (Table3; Fig.2), based on values given by the National Trust. We calculated the one-off cost of conversion of the arable farmland to wetland to be $1,110,907 ($2319 ha^−1^).

### Changes in ecosystem service values

Added together, our estimates for all of the costs and benefits of the restored wetland and arable land suggest that the overall net value of ecosystem services resulting from conversion of the arable land to restored wetland is $95,487 y^−1^ ($199 ha^−1^y^−1^) using the US Government price for carbon of $22.78 t^−1^ CO_2_ (Table3).

### Changes in beneficiaries from conversion of arable land to restored wetland

The main beneficiaries of arable land use are the relatively small numbers of local farmers who own or rent the land and the people that they employ (Table5). Compared with the arable land, twice as many people used the restored wetland for recreation, and these beneficiaries are also more widespread geographically, including small numbers of national (6%) and international (1%) visitors. Beneficiaries from climate change mitigation are global in distribution, while those from grazing and flood protection are local.

**Table 5:** Change in delivery of different services when arable land is restored to wetland, shown for beneficiaries at the local, national, and global scale. Positive symbols indicate increases, negative symbols indicate decreases, and number of symbols indicates relative magnitude of change

	Location of beneficiaries			
Ecosystem service	Local	National	Global	Level of confidence in data
*Change in annual flows if restored*				
Avoided greenhouse gas emission			+++	Low
Flood protection	+++	+		Medium
Grazing	+			High
Arable production	—	–		High
Nature-based recreation	+++	+++		Medium

## Discussion

Our study shows that for the five ecosystem services we assessed, there has been a net monetary benefit of around $95,500 y^−1^ ($199 ha^−1^y^−1^) from the conversion of arable land to wetland across the 479 ha of the restored wetland area. This estimate is based on the US Government price for carbon and increases substantially to around $193,000 y^−1^ ($403 ha^−1^y^−1^) when UK Government carbon prices are used (Table4). This estimate is based on the prices for 2011 and will necessarily fluctuate between years because of changing market prices for carbon and for services such as arable production. This might lead to smaller differences in value between the two land uses in some years. The main ecosystem services that have been gained at Wicken Fen as a result of restoration are enhanced nature-based recreation, reduced GHG emissions, increased flood protection and increased grazing by domestic stock (Table3). The main service lost after restoration is arable production. These results, however, have varying levels of confidence related to the accuracy and precision of the data (Table5), because some of the rapid techniques we used are simplified versions of well-established methods.

We omitted several services that are likely to be provided by restored wetland because we could not measure them. Perhaps most importantly and related to the original aims of the Wicken Fen Vision, we did not measure the enhancement of the wildlife value of the restoration land and its potential to buffer and make more viable the populations of rare species that occupy Wicken Fen NNR. New wetlands can reduce phosphorus and nitrogen loadings downstream through storage and recycling of nutrients (Håkanson and Bryhn 2008). Additionally, when arable land is converted to wetland, inputs of agrochemicals into surface waters and ground water (as well as GHG emissions from applying them) are reduced. Changes to water quality were not measured because no suitable inflow or outflow sites were present at which comparative measurements could be made. Ecological restoration can also lead to soil quality improvements, but we were unable to evaluate these. Likewise, we did not measure methane emissions from ditches on arable land or services such as spiritual enrichment or educational value of the restored wetland. Our overall valuation of the ecosystem services of the restoration site relative to the arable land is likely to be conservative because of these omissions.

It is also important to note that the value of nature-based recreation is unlikely to rise linearly in proportion to the area of land restored because its marginal benefit is likely to fall (Brander et al. 2006; Bateman et al. 2011). Nature-based recreation accounts for a large part of the value of the restored wetland, and thus, it is important to monitor its value over time to understand both changes in marginal benefit and the sensitivity of the overall valuation of services to this component. Results for all measured ecosystem services in this study are not necessarily applicable to other wetland restoration sites as many measurements were highly site specific (e.g., flood protection).

In a study of the value of ecological restoration on peat soils that are currently farmed in England, it is estimated that restoring existing arable land in The Fens to high, peat-forming water table conditions which exclude agriculture would provide a net value of around $2390 ha^−1^y^−1^ (£1549 ha^−1^y^−1^) ± 50–75% (Morris et al. 2010). This value is based on changes in carbon losses, GHG emissions, acidification effects of ammonia and sulfur, and in cultural services and is considerably higher than the conservative $199 ha^−1^y^−1^ reported in our study which includes a different range of ecosystem services.

A significant reason for this difference is that Morris et al. (2010) use a value of 4.20 tCO_2eq_ ha^−1^y^−1^ GHG emissions for restored land and 26.17 tCO_2eq_ ha^−1^y^−1^ for cultivated land (both on deep fenland peats) taken from Natural England (2010), compared with mid-range values of 1.69 tCO_2eq_ ha^−1^y^−1^ and 4.85 tCO_2eq_ ha^−1^y^−1^, respectively, for these two land-use types used in our study. We chose a conservative value for the land under restoration at Wicken Fen Vision because this has a very degraded peat profile, consisting of only a thin remnant, wasted peat soil over clay subsoil (Stroh et al. 2013),and we used emission factors appropriate for such soils from Bradley (1997 – cited in Natural England 2010) and Morrison et al. (2013). The estimates by Morris et al. (2010) and ours may reflect the upper and lower GWP_100_ of fenland peat under arable cultivation. It is likely that there are greater savings of emissions, particularly avoided losses of CO_2_, to be made if deeper peat residues are restored to more extensive use, than those actually restored at Wicken Fen. In a later study, Graves and Morris (2013) estimate peatland restoration to have a net value in 2012 of around £150 ha^−1^ rising to between £300 ha^−1^ and over £1000 ha^−1^ in 2080 depending on the climate change scenario measured in terms of agricultural production and carbon emissions only.

This raises the issue of the sustainability of the continuing arable use of land compared with restored wetland, which is not addressed by the data in our study. The study by Morris et al. (2010) calculates that where peat soils have wasted away, the value of land in agricultural use in the Fens drops to around US$46 ha^−1^y^−1^. Fenland peat is estimated to waste at an annual rate of 7–21 mm (Holman 2009), so that soils in the Wicken area that are often as little as 30 cm in depth will only last for 30 more years (a conservative estimate as these rates may rise with temperature increases (Davidson & Janssens 2006)). Across the fenland basin, Graves and Morris (2013) estimate that soils will last a further 30–100 years, depending on their current depth and use. On the other hand, especially where water tables can be maintained near the soil surface, restored wetlands will maintain and possibly accumulate peat (Kivimäki et al. 2008).

Neither our study nor Morris et al. (2010) include all the costs associated with drainage and pumping of water into rivers. These include the funding to drainage commissioners from central government via district authorities who levy charges on all nonagricultural properties covered by their area, and funding from the Environment Agency for water that the commissioners manage and that comes from outside their area (Middle Level Commissioners, personal communication, 12th November 2012). If these omitted costs of drainage were included, our estimates of the restoration benefits would increase.

A change in land use from arable to a restored wetland mosaic alters not only the type and value of ecosystem services generated but also the distribution of benefits (Table4). Under arable production, a small number of landowners and their employees gain the majority of the ecosystem service benefits provided by the site – as well as a sizeable direct subsidy from the taxpayer (not counted here, but worth, based on the Farm Business Survey, an estimated $177,000 y^−1^ ($370 ha^−1^y^−1^) (Rural Business Research (RBR) Farm Business Survey database 2012)). Consumers of the food produced are also beneficiaries, but restoration has only a marginal impact on this group compared with the impact on farmers for whom the arable land provides the main income. Under restoration, there is greater societal benefit to a much broader range of stakeholders, including many more local (and some long-distance) visitors, as well as the global community (through reduced greenhouse gas emissions). Yet most of these benefits do not accrue to the landowner, who (in the absence of related incentives such as carbon payments) is therefore encouraged to continue arable production rather than undertake restoration (Firbank et al. 2013).

This mismatch between private and public benefits can be reflected in political ambivalence about restoration, which may be improved by better engagement with landowners over the costs and benefits of restoration (Moss 2008). In the case of the Wicken Vision project, East Cambridgeshire District Council voted to support it in 2006, but (encouraged by a small number of local people, including some farmers) withdrew that support in 2008, before reinstating it in 2011 (East Cambridgeshire District Council 2011). We suggest that the data reported here could be used to inform this kind of debate. More generally, we hope that our approach for rapidly evaluating a broad range of services under contrasting land uses can be used to identify those of greatest benefit to society as a whole, and hence to inform a wider debate about the purpose and scope of publicly funded incentives to landowners. However, a close inspection of the transferability of values between sites is crucial so that inappropriate results are not used in these debates.

## Supporting Information

**Appendix S1.** Method for calculating global climate change mitigation. **Appendix S2.** Method for calculating nature-based recreation. **Appendix S3.** Method for calculating flood protection benefit. **Appendix S4.** Interview questionnaire for visitors, used to help calculate the value of nature-based recreation
