# Supplementary material for: Benefits and costs of ecological restoration: Rapid assessment of changing ecosystem service values at a U.K. wetland
Source: Ecol Evol. 2014 Sep 23;4(20):3875–86. doi: 10.1002/ece3.1248 (PMC4242572; doi:10.1002/ece3.1248)
Supplement: Supplementary file 1 [file ece30004-3875-SD1.doc]

Supporting Information

Appendix S1

*Method for calculating global climate change mitigation-* For restored wetland, we considered two published net carbon flux values, one for the restored wetland at Wicken Fen itself (Morrison et al 2012) and the second calculated from Levy et al (2012) (CH_4_) and Lloyd (2006) (CO_2_) from studies on the Somerset levels. The former considers only net ecosystem exchange (CO_2_ flux only) and assumes that the mean annual water table will be low enough to preclude CH_4_ emissions. Morrison et al (2012) did not measure CH_4_ fluxes at this site, but the current state of much of the new fen area, with thin and heavily mineralised peat, only fully inundated in the winter, surrounded by heavily drained arable land and vegetation communities still in flux, suggests that these emissions are likely to be low. The latter assumes that habitat restoration has raised water tables high enough to cause significant flux of CH_4_, through anaerobic decomposition. It was assumed that N_2_O fluxes are negligible because no active soil fertility enhancement is occurring. These two flux values represent the likely higher and lower global warming potential (GWP_100_)– i.e. the combined annual emission of CO_2_, CH_4_ and N_2_O over a specified timescale of 100 years after non-CO_2_ GHGs are converted to units of CO_2_ equivalent (Forster et al., 2007) – on these lands under current management. These lands are however, also grazed as part of their extensive management, though at low stocking densities, so we have accounted for GHG emissions due to grazing by ruminants, most of which are on site year round. Numbers of grazing animals on the 479ha wetland restoration area in 2011 were provided by the National Trust. The major gases produced by livestock are CH_4_ from enteric fermentation and N_2_O from manure. We accounted for these using IPCC (2006) per head values for beef cattle and horses grazing temperate grassland.

For the alternative farmland state we considered two values for peat soil oxidation under cultivation; that from Bradley (1997) (cited in Natural England, 2010) based on UK-wide values, and that of Morrison et al. (2013) measured over 120 days of lettuce production in the English Fens (we used their value, measured over 120 days to represent a minimum annual value, assuming no further emissions occurred after one crop). These represent the likely minimum and maximum published values for oxidation due to cultivation of lowland deep peat in England. We used the IPCC (2006) method for calculation of direct and indirect (leaching and volatilisation) N_2_O emissions from crop and soils, assuming 40kg per hectare inorganic nitrogen fertiliser for winter wheat and potatoes and no additional N input for oil seed rape grown on peat soils (DEFRA, 2010). The rotation present in the area shows a 71-29% split between cereals and non-cereals (Cook 2009) which we replicated in simple form as a 71%:14.5%:14.5% areal split between winter wheat, potatoes and oil seed rape. We simplified the cropping from the range of root crops and cereals quoted by Cook (2009) since these three crops are the three most grown in arable areas throughout the UK (DEFRA et al., 2014), especially on grade 1 land such as in the area around Wicken Fen. Furthermore, Tier 1 default values for calculation of N_2_O emissions from cropped land are only available for a small range of crops (IPCC, 2006).

Appendix S2

*Method for calculating nature-based recreation* - We estimated the value of nature-based recreation from the direct expenditure by visitors at and travelling to the site (see Wells 1997). The visitors consisted of local people who came to Wicken for a day-trip from nearby villages or towns or from the city of Cambridge, national tourists and international tourists. We estimated numbers of visits through a field survey of visitors to both Wicken NNR and the wetland restoration site. This gave us the ratio of visits to the restored wetland relative to those to Wicken NNR, which we combined with information from the National Trust on the annual total number of visits to Wicken NNR in 2010 to estimate the annual visit rate to the restored wetland. Surveys were conducted at the two main access points to Wicken Fen over a total of seven days in 2011 (16th, 17th, 21st, 28th August and 11th, 20th, 21st September). This survey effort aimed to sample visitors on days that were representative of the school holiday period, of school term; weekdays and weekends; and public holidays. The choice of days was made in conjunction with the National Trust who survey their visitors annually and recognise that there are different types of ‘visitor days’ based on the age, type and purpose of visitors. We focused our estimate of visits on the restored wetland only

The visitor questionnaire consisted of seven questions (for details see Appendix S4). For visitors travelling in groups, we interviewed the leader of the group wherever possible. Before the interview, we checked if the respondents had previously been interviewed to prevent double counting since some routes take visitors past both access points. We classified the visitors as local, domestic tourists or international tourists. The majority of the visitors were local either from Cambridge or the nearby towns and villages. A map depicting the NNR, the restored land and the surrounding farmland was shown to respondents to clarify the valuation area. Respondents were provided with information about the state of the current Wicken Fen Vision land before restoration (i.e. arable farmland), and about the recreation opportunities (e.g., birdwatching, dog-walking etc.) that previously existed within these areas. This was to reduce possible hypothetical bias from information effects (see Bergstrom et al. 1989). To deduce the visitor revenue under the alternative state, the visitors were asked if they would visit farmland for their chosen activities (see Appendix 1).

Our survey also asked visitors how and how far they had travelled to get to the site (which we used to estimate their expenditure on transport, A); how much they spent in the visitor centre gift shop and café (B). Hence the overall expenditure of a visitor was the travel expenditure combined with expenditure on gift and in the café (i.e. A + B). We estimated the expenditure value of recreation on arable land ($ yr^-1^) from the number of day-trippers per year to the restored wetland (C), and the proportion (D) and mean expenditure (E; in terms of $ person^-1^) of the day-trippers who would visit if it was arable farmland. The mean expenditure of day-trippers that were likely to come to the arable land ($3.76 per person) was lower than those that were unlikely to visit ($7.21 per person). Hence, estimated value of the arable land in terms of recreation = C × D × E.

Appendix S3

*Method for calculating* *flood protection benefit -* We estimated the total benefit of flood storage on the restored wetland as avoided damage to crops and property in the event of a 1 in 20 year or greater flood event that would otherwise cause lode bank failure and flooding of 2000 ha of adjacent arable land and other properties (Convine and Starling, 1988). 50 ha of this arable land would be directly affected by flooding due to failure of the lode bank system. This area, which currently consists of cereals farms (71 %) and general cropping farms (29 %) (based on Cook, 2009), would probably change to lower value sheep grazing. We estimated the benefit of this avoided flood damage to be $43,092 based on valuations in Convine and Starling (1988) and adjusted to 2011 values. The other 1,950 ha of land would be indirectly affected by higher water tables and this would lead to the replacement of general cropping farms with lower value cereal farms. The value of the avoided change in crops was estimated at $202,172, again using valuations in Convine and Starling (1988), adjusted to 2011 values. Therefore, the total cost to farmers of a flood was reckoned to be $245,264. The corresponding cost to the 10 homeowners based in the area that would be flooded was estimated at $216,241. This was derived from the Environment Agency (2010) that estimates the average damage cost of a flooded home as $20,033 based on 132,000 insurance claims associated with the summer flood events in 2007. We used this estimate, adjusted to 2011 values using inflation values, to estimate the potential total avoided damage cost for the homeowners by the restored wetland.

Therefore, the total flood protection benefit to the homeowners and farmers was calculated at $461,505. Since the embankment failure is expected to be a 1 in 20 year event, the avoided damage cost was then adjusted to $23,075 per year ($48 ha^-1^y^-1^ ) (one twentieth of the calculated total avoided damage cost)(Figure 3). No flood protection service was provided by the area under the alternative state.

Appendix S4.

*Interview questionnaire for visitors, used to help calculate the value of nature-based recreation*

Location:

Date: Time: Mode of Transport: bicycle/boat/car/horse/kayak/walk

1. If applicable, how many persons in the travel group?

Adults □ Children □

1. Where are you from? Answer: __________

NB. For domestic tourist, (1) Town/city: __________

(2) Duration of tour: __________days

(3) Type of accommodation: __________

1. Which areas will you visit?
2. National Nature Reserve (NNR)/old area of wetland habitat

Yes □ No □

1. Wicken Fen Vision Lands (WFV)/new area of wetland habitat

Yes □ No □

1. Are you likely to use the following?
2. Cafe

Yes □ No □

1. Shop

Yes □ No □

1. Have you spent/do you plan to spend money in local pubs or shops?

Yes □ No □

If yes, how much? ­­­­______________________(estimate spent as a group, if applicable)

1. What are your main reasons for visiting? (NB. If more than one reason, rank them; 1- most important)
2. Appreciating/viewing nature and/or wildlife □
3. Exercise, sports or hobbies (include horse-riding, dog walking) □
4. Time with family or friends □
5. Others­ ______________________________ □
6. Would you come for these activities if Wicken Fen (all areas) was farmland?

Yes □ No □
